# Multiple Nuclear Gene Phylogenetic Analysis of the Evolution of Dioecy and Sex Chromosomes in the Genus *Silene*

**DOI:** 10.1371/journal.pone.0021915

**Published:** 2011-08-10

**Authors:** Gabriel A. B. Marais, Alan Forrest, Esther Kamau, Jos Käfer, Vincent Daubin, Deborah Charlesworth

**Affiliations:** 1 Laboratoire de Biométrie et Biologie évolutive, UMR5558, Université Lyon 1, Centre National de la Recherche Scientifique, Villeurbanne, France; 2 Institute of Evolutionary Biology, University of Edinburgh - King's Buildings, Edinburgh, United Kingdom; Montreal Botanical Garden, Canada

## Abstract

In the plant genus *Silene*, separate sexes and sex chromosomes are believed to have evolved twice. *Silene* species that are wholly or largely hermaphroditic are assumed to represent the ancestral state from which dioecy evolved. This assumption is important for choice of outgroup species for inferring the genetic and chromosomal changes involved in the evolution of dioecy, but is mainly based on data from a single locus (ITS). To establish the order of events more clearly, and inform outgroup choice, we therefore carried out (i) multi-nuclear-gene phylogenetic analyses of 14 *Silene* species (including 7 hermaphrodite or gynodioecious species), representing species from both *Silene* clades with dioecious members, plus a more distantly related outgroup, and (ii) a BayesTraits character analysis of the evolution of dioecy. We confirm two origins of dioecy within this genus in agreement with recent work on comparing sex chromosomes from both clades with dioecious species. We conclude that sex chromosomes evolved after the origin of *Silene* and within a clade that includes only *S. latifolia* and its closest relatives. We estimate that sex chromosomes emerged soon after the split with the ancestor of *S. viscosa*, the probable closest non-dioecious *S. latifolia* relative among the species included in our study.

## Introduction

Many plants with separate sexes (dioecy) evolved this sex system much more recently than the main animal model systems (mammals, *Drosophila* and birds). Plants such as *Silene latifolia*, *Carica papaya*, *Bryonia dioica* and *Fragaria* may thus be suitable for studying the first steps in sex chromosome evolution [1], [2], [3].

*Silene latifolia* and its dioecious close relatives, *S. dioica*, *S. marizii*, *S. heufellii* and *S. diclinis* [4], have an XY chromosomal sex determination system. In a distinct group of dioecious species with no chromosome heteromorphism, *S. otites*, *S. colpophylla* and *S. acaulis* which are closely related (see [5] and our results below), separate sexes probably evolved independently [4], [6], [7]. Many other *Silene* species are non-dioecious. These are either hermaphroditic, or else gynodioecious (with some individuals hermaphroditic and others female), or gynodioecious with gynomonoecious individuals (having both hermaphroditic and female flowers). Dioecy in *Silene* therefore probably evolved from hermaphroditism via gynodioecy (rather than from monoecy, a common ancestral state for dioecious plants, in which individuals have separate male and female flowers [8], [9], [10], [11]).

Evolution of dioecy from hermaphroditism via gynodioecy requires at least two mutations [12]: first a male-sterility mutation creates females and establishes a polymorphism for females and hermaphrodites (gynodioecy), and the hermaphrodite individuals (with male function) may then evolve increased maleness through partial or complete female-sterility mutations. Because the second mutation, suppressing female functions, lowers the reproductive fitness of the females carrying the first mutation, linkage is required between the genes that undergo the mutations [12]. This predicts that a single autosome in an ancestral species evolves into a proto-sex chromosome pair (as opposed to chromosome rearrangements bringing the sex-determining genes together from different ancestral chromosomes to form a non-recombining region carrying the male- and female-sterility factors inferred genetically, see [12], [13]). The initial linkage may be followed by selection for reduced genetic recombination in the sex-determining region. Cessation of recombination can occur in a stepwise manner, beginning in the region flanking the sex determining locus and then spreading to other parts of the sex chromosomes, leading to the large non-recombining genome regions now found on many sex chromosomes, including those of *S. latifolia* [13], [14].

Once recombination stops between a gene or region on the sex chromosome pair, sequences in the region start diverging, and sequence divergence can indicate the age of the sex chromosome system. In *S. latifolia*, divergence has been estimated between multiple loci spread across the X and Y chromosomes. The maximum nucleotide sequence divergence between X and Y chromosomes is ∼20%, suggesting that recombination stopped in the oldest such region between 5 and 10 MYA [15], [16].

This X-Y sequence divergence is similar to the estimated divergence for the same loci between *S. latifolia* and some non-dioecious *Silene* species, including the gynodioecious *S. vulgaris* [17], [18] and the hermaphrodite *S. conica* [19]. The estimated date of the split of *Lychnis* and *Silene* from outgroups is ∼12.4 MY ago, and that between the different *Silene* subgroups 7.9–9.5 MY [20], similar to the date estimated above for initiation of X-Y divergence. The possibility must thus be considered that sex chromosomes in *Silene* evolved early in the evolutionary history of the genus, and could have been secondarily lost in ancestors of the non-dioecious *Silene* species. Under the evolutionary model for dioecy outlined above, a single mutation can cause reversion to gynodioecy or to full hermaphroditism (through loss of a major female-sterility mutation). It is therefore important to test whether dioecy is ancestral (and hermaphroditism a reversion from this) in plant taxa used to study early stages of sex chromosome evolution.

In *Silene*, it is important to consider the possibility of reversion to hermaphroditism, because reversion is probably common in plants [21], and reverted species are unsuitable as outgroups for inferring directions of changes. For example, the inference that a single pair of autosomal chromosomes in an ancestral *Silene* species evolved into a sex chromosome pair (as the population genetics outlined above predicts) is based on the mapping of homologues of genes in the non-recombining regions of the Y chromosomes of *S. latifolia* to a single *S. vulgaris* autosome [22]. However, if *S. vulgaris* had a dioecious ancestor, it could be incorrect to infer that the sex chromosomes evolved without translocations from a state like that in *S. vulgaris*. Similarly, one could not infer the ancestral states at orthologues of sex-determining loci by using the hermaphrodite *S. viscosa* [23].

The only sequence-based study available that is relevant for selecting outgroups for studying the origin of dioecy in *Silene* used the internal transcribed spacer of the ribosomal DNA (ITS regions) in the nuclear genome, and included 25 *Silene* species [4]. This has remained the accepted phylogeny relevant for the evolution of dioecy in *Silene*, and is consistent with evolution of different sex-determining genes in *S. colpophylla* (a relative of *S. otites* and *S. acaulis*), based on the finding that its orthologues of 4 sex-linked *S. latifolia* genes are in one *S. colpophylla* linkage group, but are not sex-linked [24].

However, such phylogenies based on a single genomic region represent only that region's evolutionary history, which may differ substantially from that in other genome regions, and may not represent the species' ancestry. Other phylogenetic work on *Silene* has added chloroplast sequences and some nuclear sequences, including likely single-copy genes [25], but mainly examined deeper relationships in the family Caryophyllaceae, often including few members of one subgenus when studying the phylogenetic relationships of another subgenus [25], [26], [27], [28], [29], [30], or have focused on a particular group [7], [31], rather than including all species relevant for the study of the evolution of sex-determination within *Silene*. New analyses of DNA sequences are therefore needed, ideally using multiple unlinked loci. Here we report the first multi-gene study in *Silene*, including both sections with dioecious species, *Melandrium* and *Otites* [20], [32]. Our taxonomic sampling also includes other *Silene* species from subgenera *Behen* and *Silene* (in the terminology of [25]), *Lychnis* and, as an outgroup for rooting trees, we used a *Petrocoptis* species [20], [25]. Our inclusion of suitably selected species to help infer the relationships, enabled us to use BayesTraits analysis to infer the evolution of dioecy in a phylogenetic context that allows for reversals.

We used nuclear genes, because sequences from the cytoplasmic genome lack frequent recombination, and do not yield independent phylogenies. In *Silene* as in other plants, cytoplasmic genomes introgress more frequently than nuclear genes [20], which blur true phylogenetic relationships. Also, transpositions of large regions of chloroplast sequences to the *S. latifolia* Y chromosome [33], and perhaps to other chromosomes, can confuse phylogenetic inferences. Furthermore, in *Silene* these genomes may have had major changes in mitochondrial mutation rates [34], [35], experienced complex mutational processes, and/or have been under positive selection [36], [37], [38]. To deal with the difficulties of data from multiple, independently evolving genome regions, we employed several recently developed approaches for multi-gene phylogenetic analysis.

## Materials and Methods

### Plant material

Following [4] we chose fourteen species representing the diversity of breeding systems within *Silene* (see Table S1, which also gives voucher details and GenBank accession numbers of the genes sequenced). The 14 species include *Lychnis coronaria* and *L. flos-jovis* as *Lychnis* appears as a close sister group to *Silene* in the chloroplast+ITS phylogeny [20]. We also sequenced the genes from either *Petrocoptis hispanica*, a close outgroup species of *Silene*+*Lychnis* [25], [29], [30] or *Dianthus* (for the *PGK* gene, which did not amplify from *P. hispanica*). Plants were grown from seeds collected in the wild, or obtained from the Royal Botanic Garden Edinburgh's wild collections.

### Molecular methods

Genomic DNA was extracted from young leaves using the FastDNA kit (Q-BioGene). We sequenced 10 autosomal genes and 3 genes that are sex-linked in *S. latifolia*, *S. dioica* and *S. diclinis* (Tables S2 and S3). The autosomal genes were chosen from *S. latifolia* ESTs [39]. The putative functions of the loci newly sequenced for this study (*ABCtr*, *ATUB*, *ADPGph*, *ClpP3*, *ELF*, *LIP21*, *OxRZn*, *PSIcentII*, *PGK* and *2A10*) are listed in Table S2, together with the primers used for sequencing, and the PCR conditions. The sex-linked loci sequenced were *SlXY4* [40], *SlXY7* [16], and *SlXYCyp* [16]. Intron-exon boundaries were determined by alignment with cDNA sequences of *A. thaliana* homologues, to avoid primer sites spanning introns; most sequences include coding regions and introns.

### Sequencing and alignment

Sequences were obtained by direct sequencing of PCR products after removal of excess primers and unincorporated dNTPs using shrimp alkaline phosphatase and *Exo*I respectively (ExoSAP-IT; Amersham Biosciences). Both strands were sequenced using BigDye Cycle Terminator protocols (Amersham Biosciences) on an ABI377 automated capillary sequencer. Whenever we found more than one sequence in autosomal genes, or when the primers amplified both X and Y copies of sex-linked genes, PCR products were cloned into TOPO TA Cloning kits (Invitrogen) and sequenced as described above.

To detect and exclude paralogues (which can confuse estimates of species relationships), direct sequences were obtained from several plants for as many species as possible; for dioecious species, DNA was sampled from both male and female plants (except for *S. heuffelii*, for which only one male was available). The sequences often included heterozygous sites, and, from such individuals, both sequences were determined after cloning. Sequences for each gene were inspected using Sequencher version 4.0.5 (Gene Codes Corporation) and aligned using Clustal X version 1.81 [41] using the default parameter values. When each species had <1% divergence between the sequences amplified with a set of primers, the sequences were assumed to be alleles of a single-copy locus. Loci not satisfying this criterion were discarded (in total, about the same number of loci as the number analysed, data not shown). The alignments were modified manually using BioEdit version 7.0.4.1 [42]. Poorly aligned positions were removed using Gblocks [43] with the “relaxed block selection” option [44]. The resulting alignments total 5582 bp (Table S3 shows the numbers of sites per gene). ITS1 and ITS2 sequences were retrieved from GenBank (1503 bp, or 1195 bp after Gblocking).

### Concatenation of genes

When several sequences were available for a given gene in a species, we used the software ScaFos to select the sequence from the alignments after GBlocking (see above), the one with the smallest average divergence from the other conspecific sequences [45]. Fast evolving species were not excluded, and we used minimal evolutionary distances with a gamma distribution, with maximal 50% missing sites (the other options used were: minimal length to select a sequence = 0%, making chimera = YES). We then used Concaterpillar, a hierarchical clustering method based on likelihood-ratio testing that identifies genes with congruent phylogenies, and very efficiently identifies loci that can be concatenated for estimating phylogenies [46].

### Phylogenetic tree reconstruction

Individual locus trees were constructed using PhyML [47], [48] with the General Time-Reversible (GTR) model, the estimated base frequencies, percentage of variants and alpha parameter for the gamma distribution, and with 4 or 8 rate categories. We used the GTR+Γ as it is the most sophisticated model available for nucleotide sequences but other simpler models gave the same conclusions (data not shown). Support for nodes was evaluated with 100 replicates of non-parametric bootstrapping, again using the maximum likelihood optimality criterion. For the sex-linked loci, only X sequences were included; Y sequences were not used for the phylogeny, because of their unusually fast sequence evolution [49], [50], [51].

**Figure 1:**
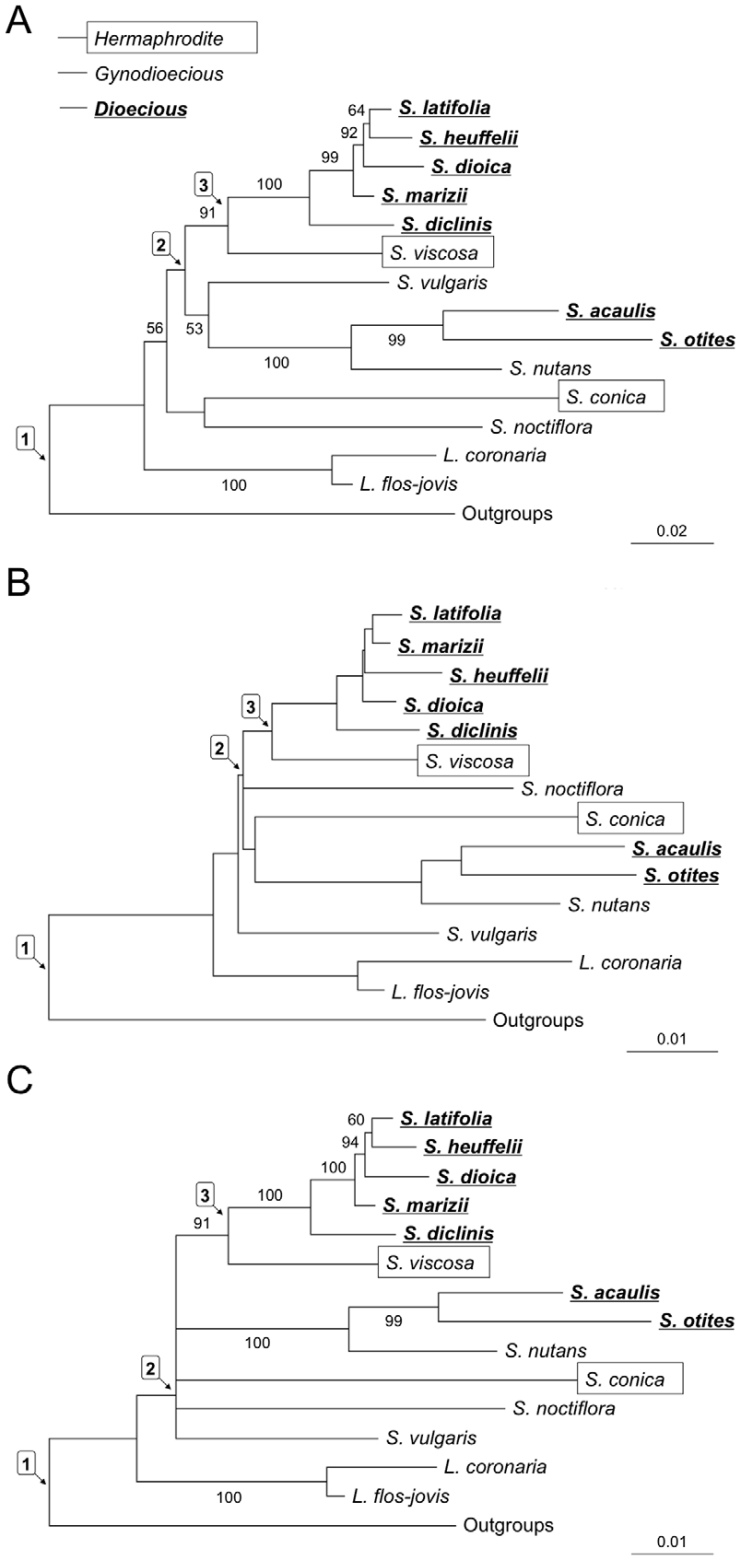
Phylogeny of the *Silene* species studied. Only bootstrap values >50% are shown. The breeding systems of the species are indicated in the figure. The outgroup for rooting the tree for the genus *Silene* was usually *P. hispanica* (see Tables S2 and S3 for details). For the sex-linked genes, only X sequences were included. **A**) Maximum likelihood tree obtained with PhyML using the concatenated alignment with 12 genes (*LIP21* and the outgroup sequences for *2A10*, *clpP3*, *ELF* were excluded by Concaterpillar). **B**) Dataset is as in A, but tree obtained by combining all 12 gene trees using the SDM method. **C**) Consensus tree built from the trees in A and B. Labels 1, 2 and 3 in the trees indicate the nodes used in the BayesTraits analysis (see main text).

We used three different approaches to combine the results from different genes. First, we estimated PhyML trees using concatenated sequences (with the parameters above). By default, we used GTR+Γ. For the dataset used to build the tree shown in Figure 1A, we tested the effect of the models for sequence evolution. jModeltest [52] recommends the “transitional model”+invariant+gamma model (TIM+I+Γ) and the Tamura-Nei+invariant+gamma model (TrN+I+Γ) as the best of the 88 models tested for the sequences using respectively the Akaike and Bayesian information criteria (AIC and BIC). We thus built the tree shown Figure 1A using TrN+I+Γ (best model using BIC criterion, see [53]), which is implemented in the PhyML. Note however that the topology of this tree is not affected by model selection since TrN+I+Γ, GTR+Γ, or the model-averaged option available in jModelTest give identical topologies.

Second, we built supertrees from the individual gene trees using the Super Distance Matrix (SDM) method, which normalises tree lengths before combining the trees; this is less sensitive to rate heterogeneity than the first method [54]. We then imported the supermatrix of squared distances into BIONJ [55]; this method does not yield bootstrap values.

Genes with trees that differ from the species tree can produce misleading species trees when the latter is estimated from concatenated genes [56]. Methods incorporating an explicit model of coalescence [57] have been developed specifically to deal with deep coalescence. To allow for lineage sorting and other population genetic events that can result in different genes having incongruent trees (e.g. [58], [59]), we therefore also analyzed the multi-locus data using a third approach, available through the R package phybase [60]. As explained in Text S2, this includes two methods. STAR is based on the average ranks of coalescence events, and STEAC employs average coalescence times [61].

Tree topologies were compared by the Approximately Unbiased (AU) test, using Treefinder [62].

### Analysis of character evolution

We analysed the evolution of breeding systems in *Silene*, following [63] with the BayesMultiState function in BayesTraits [63]. Breeding system data followed [4]. For the two *Lychnis* species, *S. noctiflora* and *S. acaulis*, we allowed two possible character states, because for these species both gynodioecy and hermaphroditism are recorded (*Lychnis* and *S. noctiflora*) or gynodioecy and dioecy (*S. acaulis*) [64], [65], [66]. The results were not qualitatively affected by allowing two character states, or only one (data not shown). We used maximum likelihood to estimate the probabilities for three different states, dioecy, gynodioecy and hermaphroditism, at several relevant nodes, and the model allowed transitions and reversals between each of these states.

### Distribution of pairwise divergence values

To compute the *d*_S_ values for *S. latifolia* from other species, or X-Y *d*_S_ values for *S. latifolia* sex-linked genes, we extracted the coding regions of each gene and used codeml in PAML [67]. The mean *d*_S_ values for each comparison of *S. latifolia* with other groups of species were compared using a non-parametric sign-test with setting either 0.06 (*d*_S_ value for XY_2_) or 0.16 (*d*_S_ value for XY_1_) as theoretical values. The test is significant when the null hypothesis (the number of observed *d*_S_ values>the theoretical value = the number of observed *d*_S_ values<the theoretical value) is rejected.

## Results and Discussion

### The phylogenetic relations of the *Silene* species studied

Several trees from individual genes show the expected grouping together of *S. latifolia*, *S. dioica*, *S. marizii*, *S. heufellii* and *S. diclinis* (6/13 have significant bootstrap support, and 2 have some bootstrap support, see Figure S1). *S. acaulis* and *S. otites* (and the gynodioecous species *S. nutans*) also often form a clade (6/13 trees), as also do the two *Lychnis* species (7/13 trees). However, the topologies and relationships among these three clades differ among the individual loci. We therefore used several approaches to estimate the species tree using combined data.

We first used concatenated sequences. To test whether concatenating different gene sequences is appropriate, we used Concaterpillar. This requires the same set of species for all the loci, limiting our analysis to only 11 of the 14 ingroup species, and only 9 genes, including the ITS sequences (Table S3). However, excluded species (*S. nutans*, *S. heuffelii*, or *S. marizii*), are all very close relatives of included ones, and their exclusion had little effect on the results. With this reduced dataset, the *LIP21* gene falls into a distinct block (top part of Table S4, block 3). Additionally excluding outgroup sequences for *2A10*, *clpP3*, *ELF*, Concaterpillar recovered a single block with the 7 newly sequenced autosomal genes together with ITS (lower part of Table S4, block 1). This concatenation confirms the early divergence of *Lychnis* relative to the species included in the present study [25], [29], [30], and also the distinctness of the two dioecious clades inferred using ITS (Figure S2 and Text S1). Also *S. latifolia* and its close dioecious relatives (*S. dioica*, *S. diclinis*, *S. heuffelii*, *S. marizii*) are a monophyletic group, as in the individual gene trees. However, sequence information from these species was probably lost in removing poorly aligned regions, in order to include all the species (see Methods). Because this Gblock pre-processing is unnecessary for such close relatives, we also analysed the raw alignments of just these species. Using maximum likelihood (Figure S3), we get slightly different relationships among the dioecious species, which are, however, fully consistent with those in the trees presented below in Figure 1.

A PhyML tree using a concatenation of the Concaterpillar block of 7 non-ITS sequences described above, plus the 5 other genes (Figure 1A) yields the same statistically supported clades as just the 7 genes, and the bootstrap values are as good. *S. viscosa* is now closest to the *S. latifolia* group species (although statistical support is weak), and *S. vulgaris* more distant. This is consistent with genomic *in situ* hybridization results [68], and is biologically plausible, because *S. viscosa* (unlike *S. vulgaris*) can hybridize with *S. latifolia*, suggesting a close relationship [23]. However, a chloroplast tree [20] suggests a closer relationship for *S. vulgaris* than *S. viscosa*. In that tree, as in ours, *S. conica* no longer appears to be a close relative of *S. latifolia*, a major difference from previous conclusions based on few genome regions. Identical results were found with 4 or 8 site categories, and also using RaxML [69] with 25 site categories and Treefinder with various optimization methods (data not shown), and also with other models of sequence evolution (see Methods). The SDM tree combining the 12 gene trees (Figure 1B) is similar to the PhyML tree.

Species trees using STAR or STEAC (Figure S4 and Text S2) are very similar to Figure 1B and are fully consistent with the consensus tree shown in Figure 1C. These methods are based on a coalescent model, and can thus handle incongruencies due to incomplete lineage sorting, which can adversely affect concatenation-based methods [56]. Moreover, as they use either average coalescence times (STEAC) or ranked coalescence times (STAR), the results are not biased by missing data. Finally, the rank-based STAR method is robust against differences in evolutionary rates or branch length [61].

### The evolution of dioecy

We ran BayesTraits using topologies in Figures 1A and 1B, which were based on the alignment of the 12 genes, and do not differ significantly (AU test; *p* value: 0.995), and on a consensus topology built from these trees using Treefinder (Figure 1C). In all analyses, dioecy is unlikely at deep nodes (nodes 1 and 2 in Table 1). Gynodioecy or hermaphroditism are the most probable ancestral states for *Silene*, and the results for node 2 support the belief that dioecy evolved at least twice in the genus. Our results for node 3 suggest, with less certainty, that dioecy evolved after the split of *S. latifolia*, and its close dioecious relatives from the common ancestor of *S. latifolia* and *S. viscosa* (see Table 1).

**Table 1:** Probabilities of dioecy and other sexual systems at three critical nodes in the *Silene* phylogeny, estimated using BayesTraits.

Node and description of the node	Phylogeny used in the analysis	Probability dioecious	Probability gynodioecious	Probability hermaphrodite	Probability non-dioecious
**Node 1**	Figure 1A	<10^−4^	0.457	0.543	>0.999
Root of the tree	Figure 1B	0.240	0.388	0.372	0.760
	Figure 1C	<10^−4^	0.488	0.512	>0.999
**Node 2**	Figure 1A	<10^−4^	0.687	0.313	>0.999
Common ancestor of all dioecious species,	Figure 1B	0.488	0.198	0.313	0.511
including *otites* and *latifolia* groups	Figure 1C	<10^−4^	0.540	0.460	>0.999
**Node 3**	Figure 1A	<10^−4^	0.478	0.522	>0.999
Common ancestor of the *latifolia* group and	Figure 1B	0.623	0.115	0.262	0.377
*S. viscosa*	Figure 1C	0.113	0.401	0.486	0.887

Two possible character states were allowed for *Lychnis*, *S. noctiflora* and *S. acaulis* (see text).

It seems unlikely that our sparse sampling of *Silene* species could lead to erroneous inference of the ancestral character state. Fundamental relationships within *Silene* are well enough established [25], [30], [70] to be confident that *Lychnis* is basal to the species studied here, and that the two major subgenera (*Behen* and *Silene*) within our set of *Silene* species each includes one of the two clades of dioecious species discussed above [4], [20]. We included in our analysis species from both these major *Silene* clades, as well as suitable outgroups. Many other species that were not included in our analysis are however, known to be hermaphrodites [4]. Thus, our sample over-represents dioecious species, which is conservative, because any bias would be towards inferring a dioecious ancestor.

Furthermore, simulations of taxon sampling [71] show that it is more important to include basal species (as in our sample) than many late-diverging species; when a molecular clock is not applicable, adding more taxa can even decrease the quality of inference (in our trees, some differences in branch lengths are large, see Figures 1, S2 and Text S1). When a molecular clock is assumed, adding more taxa increases the probability of inferring the correct state for the ancestor, but not greatly, and improvements occur mainly when the character changes rapidly [72]. If there are fewer character changes over the ancestry than assumed in these models, as is probably true in *Silene*, given the fairly short time-scale of evolution within the genus, and the low likelihood that dioecy will evolve, due to the need for at least 2 genetic changes (see Introduction), the effect is minor, even when as few as 16 out of more than 500 taxa are sampled [72]. Therefore, although we included only 14 *Silene* species out of 700 estimated in the genus [26], [27], [32], [73], [74], this should not be a serious problem, particularly if the species omitted are mostly hermaphrodites or gynodioecious, although including more taxa would, of course, change the probabilities in Table 1.

### The phylogeny of sex-linked genes and the evolution of sex chromosomes

**Figure 2:**
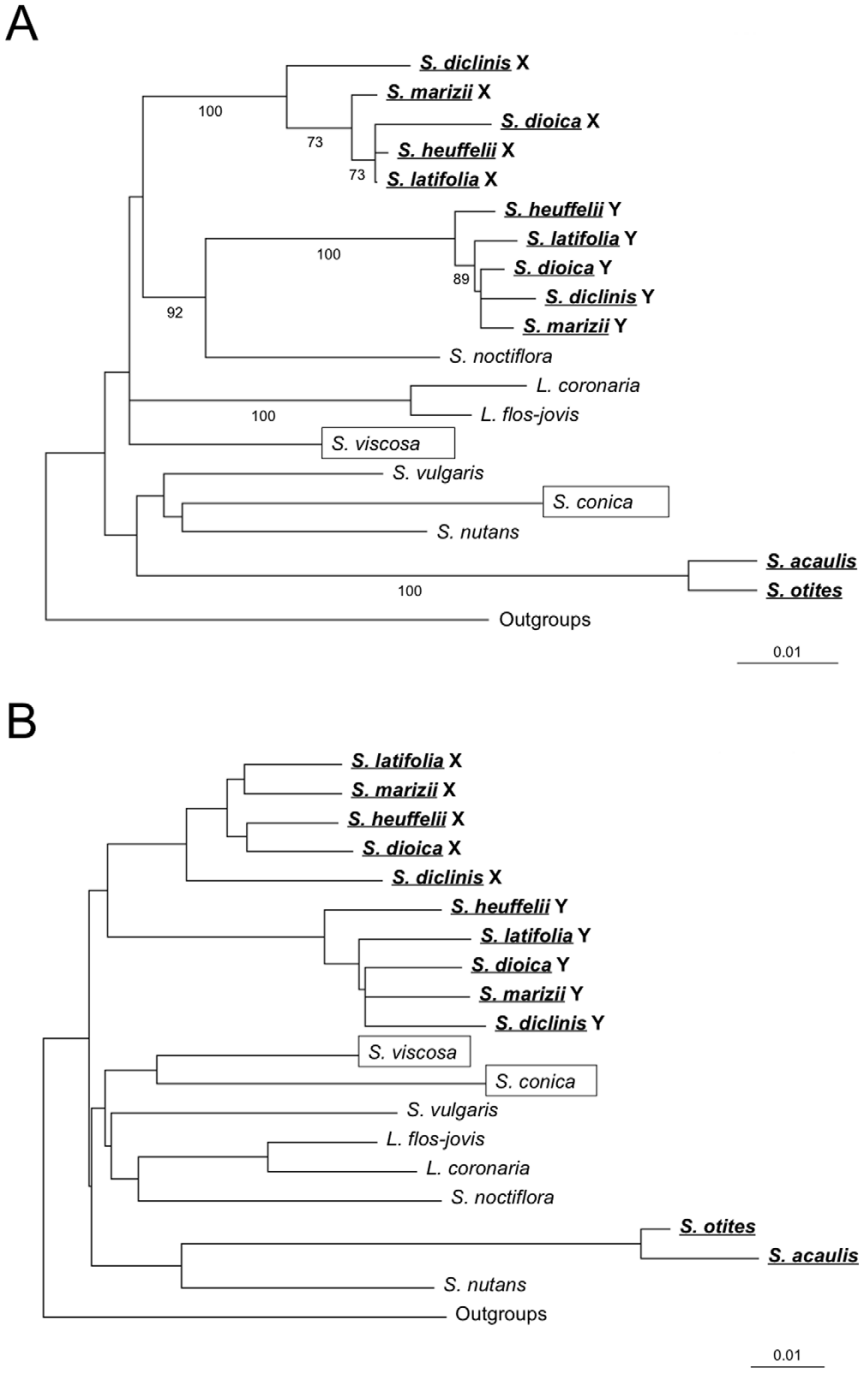
Concatenate tree and supertree for sex-linked genes. **A**) PhyML tree (GTR with gamma distribution) for the concatenated alignments of the three sex-linked genes. Bootstrap values >50% are shown. **B**) SDM tree from the three individual sex-linked gene trees.

To independently test for an early emergence of dioecy in *Silene*, followed by loss of dioecy in some taxa, we also analysed X and Y sequences of the three genes that are sex-linked in *S. latifolia* and the four closely related dioecious species. If the sequences of a gene include a monophyletic X and Y group of sequences, we can infer that sex chromosomes evolved in the lineage ancestral to the *S. latifolia* group of dioecious species. Genes estimated to have stopped recombining soon after the sex chromosomes first evolved [15], [16] are the most relevant; of these, the *SlXY7* tree is fully consistent with the combined data, whereas the *S. otites* sequences of the *SlXY4* homologue branch with the Y-linked sequences, but non-significantly (AU test *p* = 0.563, see also Figure S1). The concatenation and supertree approaches using the whole X and Y sequence dataset also support this conclusion. The SDM tree (Figure 2B) also supports evolution of the sex chromosomes after the origin of the genus. In the PhyML tree (Figure 2A), *S. noctiflora* branches with the Y sequences, but this topology is not significantly better than one with X and Y sequences forming a monophyletic group (AU test p = 0.136).

### Comparing the age of *S. latifolia* sex chromosomes with species divergence times within *Silene*

**Figure 3:**
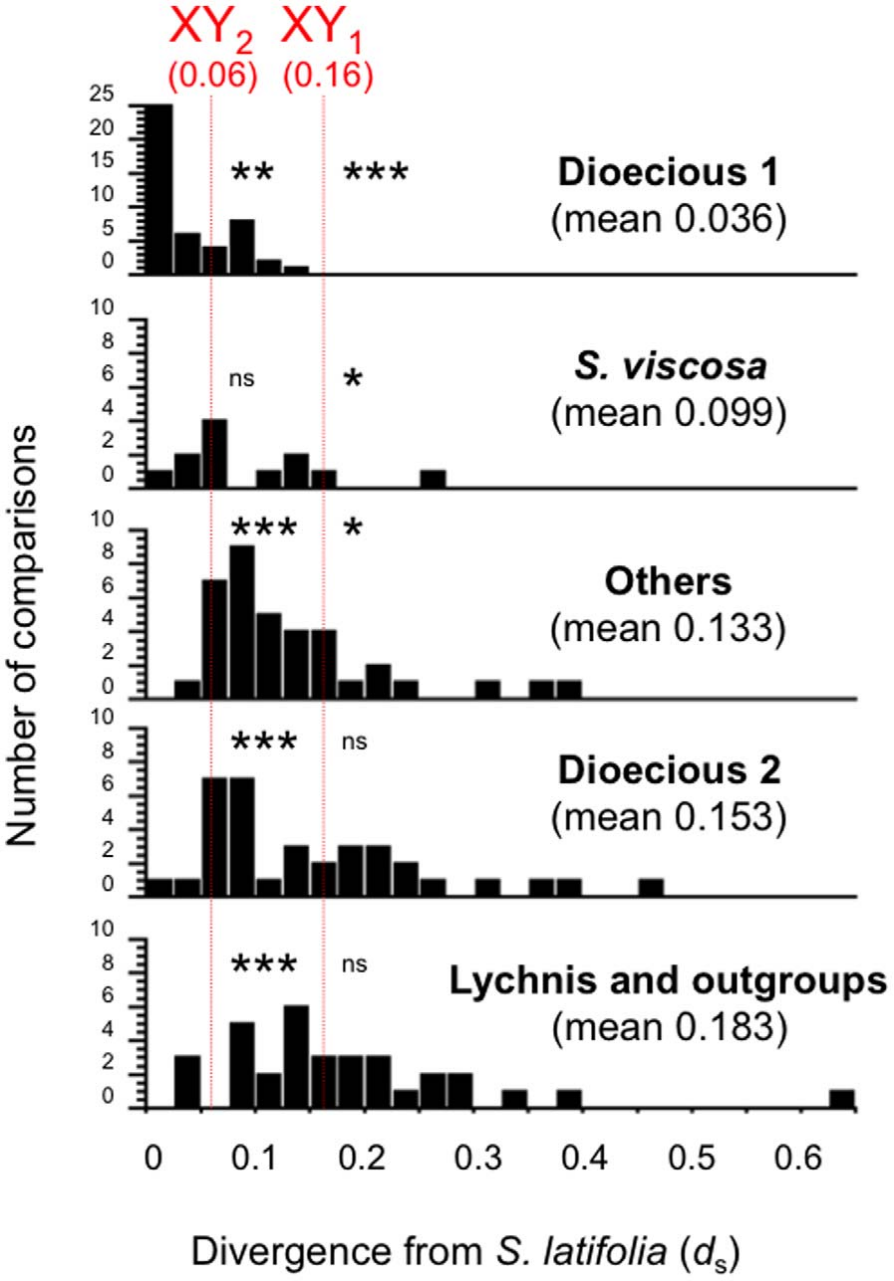
Divergence between sex chromosomes and between *Silene* species. Distribution of synonymous divergence values (mean *d*_S_ values, estimated using PAML) from pairwise comparisons between *S. latifolia* and the other species for the 13 genes. One such comparison is for *S. viscosa*, and the others are for the following groups of species: Dioecious 1 = *S. dioica*, *S. diclinis*, *S. marizii*, *S. heuffelii*, Dioecious 2 = *S. otites*, *S. acaulis*, *S. nutans* (although, as noted in the text, most *S. nutans* populations are gynodioecious), Others = *S. vulgaris*, *S. conica*, *S. noctiflor*a, *Lychnis* and outgroups = *Lychnis flos-jovis*, *Lychnis coronaria* and outgroups. In addition, vertical arrows indicate the average *d*_S_ between the *S. latifolia* X and Y sequences for *SlXY4* and *SlXY7*, which belong to the oldest stratum of X-Y divergence (symbolised by XY_1_), and for *SlCyp-*XY (labelled XY_2_), a gene which belongs to the “intermediate stratum” that stopped recombining considerably later than the first two genes [15], [16], [75]. For each panel in Figure 3, we performed a sign-test (see Methods) of whether either the XY_1_ or the XY_2_ *d*_S_ value is significantly different from the mean in the panel (e.g. for the top panel, we tested *S. latifolia* vs. Dioecious 1). Significant differences are indicated as follows: * p<10^−1^, ** p<10^−2^, *** p<10^−3^; ns = non-significant differences.

Finally, if the sex chromosomes in *S. latifolia* (plus its closest relatives) evolved within the genus, divergence between *S. latifolia* and most of the species in the genus should exceed the maximum X-Y divergence. As another test, we therefore computed pairwise synonymous divergence values (*d*_S_) between *S. latifolia* and the other species sampled, using only X-linked sequences for the *S. latifolia* sex-linked genes, and compared them with X-Y *d*_S_ values for three *S. latifolia* sex-linked genes, *SlXY4*, *SlXY7* and *SlCyp-XY* (Figure 3). Using a non-parametric sign-test (see Methods), we show in Figure 3 that the average X-Y *d*_S_ value for the most highly diverged sex-linked genes, *SlXY4* and *SlXY7* [15], [16], [75] (i) is, as expected, significantly higher than the values between the close dioecious relatives of *S. latifolia*, (ii) is marginally significantly higher than the value from *S. viscosa* and also *S. vulgaris*, *S. conica* or *S. noctiflora*, and (iii) does not significantly exceed the higher divergence between *S. latifolia* and the other group of dioecious species (*S. otites* and *S. acaulis*), or the *Lychnis* species (or the outgroups). Overall, these results are consistent with an origin of sex chromosomes within an ancestor of *S. latifiolia* and its close dioecious relatives, rather than the alternative of an earlier origin. As expected, the divergence of *SlCyp*-X and -Y started much more recently [15], [16], [76], and our analysis places this event long after the split from the *S. otites* group of dioecious species, and probably after the split from *S. viscosa* (although the test in this case is non-significant). We find no clear evidence for reversions from dioecy to hermaphroditism or gynodioecy among dioecious species related to *S. latifolia*. The date when dioecy evolved in the ancestor of *S. otites* remains unknown, but it should be possible to estimate this date once the sex-determining chromosome of these species is identified.

## Supporting Information

Figure S1 **Individual gene trees for our 13 genes.** Trees were obtained with PhyML (see Material and Methods) with 100 non-parametric bootstrap replicates (only values >50% are shown). Note that some of the sequences were excluded from the further analyses, as follows: cDNA sequences from *S. latifolia* for *ABCtr*, *PGK*, *SlXY4* and *SlXYCyp*, whose sequence lengths were too different, and the *Petrocoptis* sequence of *OxRZn* because of doubts of the orthology of the sequence. For each gene, there is one outgroup sequence. As noted in Table S2 and footnote 4 of Table S3, the outgroup species used to root the tree is usually *Petrocoptis hispanica*; this sequence was the outgroup for 8 of the 13 trees shown, including all the X-linked genes, but, for *PGK*, a *Dianthus* sequence was used, and *Lychnis* for *ATUB*-A and *OxRZn*.
(TIFF)

Figure S2 **Other inferred phylogenies of the** ***Silene*** **species studied.** For details about sequences and species, see Table S3. Only bootstrap values >50% are shown. **A**) Maximum parsimony tree of the ITS sequences from 12 species from [4] that were also studied by ourselves. The different mating systems are indicated, and stars indicate bootstrap values larger than 50%. **B**) Maximum likelihood tree (PhyML) of the ITS sequences from 13 of our species. **C**) Maximum likelihood tree of 7 autosomal genes concatenated by Concaterpillar (see Table S4) with all the species (except outgroups for 3 genes: *2A10*, *ClpP3* and *ELF*, see main text and Table S4) obtained with PhyML. Identical results were found with 4 or 8 categories of sites. In trees **C** and **D**, and also those in Figure 1 of the main text, only X sequences were included for the sex-linked genes. **D**) Dataset as in C, but the tree was obtained by combining all the 7 gene trees using the SDM method. In **C**, *S. conica* and *S. noctiflora* diverge early, but in **D** they group with *S. vulgaris* and *S. viscosa*.
(TIFF)

Figure S3 **Tree for dioecious species, rooted using** ***S. diclinis*** **(as this species is early diverging in all trees in** **Figure 1** **and Figure S2).** The tree was obtained by PhyML on the concatenate of all genes without Gblocking. Values indicate the results of non-parametric bootstrapping with 100 replicates (only values >50% are shown).
(TIFF)

Figure S4 **Estimated species trees, using all genes except for** ***LIP21*** **(12 loci).** A) Tree using STAR. B) Tree using STEAC. In both cases, the branch lengths are proportional to the bootstrap support, which is given as a percentage at each branch. As for the individual locus trees, the outgroup species was *Petrocoptis hispanica* for all genes except *PGK*, where a *Dianthus* sequence was used, and *ATUB-A* and *OxRZn* where no outgroups were available.
(TIFF)

Table S1 **Provenance and voucher details of plants used to assess autosomal and sex-linked gene phylogenies in** ***Silene*** **.**
(DOC)

Table S2 **Primers used to amplify genomic DNA of the genes studied, lengths of the sequences studied, and the outgroup used.**
(DOC)

Table S3 **List of species and sequences.**
(DOC)

Table S4 **Results of tests for combining alignments using Concaterpillar.**
(DOC)

Text S1 **Discussion on the differences in branch length in our trees.**
(RTF)

Text S2 **Methods and results for the STAR and STEAC analysis.**
(RTF)

Reference S1 (RTF)

## References

[1] Charlesworth D, Guttman DS, Ainsworth CCe (1999). The evolution of dioecy and plant sex chromosome systems.. Sex Determination in Plants.

[2] Liu Z, Moore PH, Ma H, Ackerman CM, Ragiba M (2004). A primitive Y chromosome in Papaya marks the beginning of sex chromosome evolution.. Nature.

[3] Spigler R, Lewers K, Main D, Ashman T-L (2008). Genetic mapping of sex determination in a wild strawberry, *Fragaria virginiana*, reveals earliest form of sex chromosome.. Heredity.

[4] Desfeux C, Maurice S, Henry JP, Lejeune B, Gouyon PH (1996). Evolution of reproductive systems in the genus Silene.. Proc Biol Sci.

[5] Eggens F, Popp M, Nepokroeff M, Wagner W, Oxelman B (2007). The origin and number of introductions of the Hawaiian endemic *Silene* species (Caryophyllaceae).. American Journal of Botany.

[6] Mrackova M, Nicolas M, Hobza R, Negrutiu I, Monéger F (2008). Independent origin of sex chromosomes in two species of the genus *Silene*.. Genetics.

[7] Frajman B, Eggens F, Oxelman B (2009). Hybrid origins and homoploid reticulate evolution within Heliosperma (Sileneae, Caryophyllaceae) — a multigene phylogenetic approach with relative dating.. Systematic Biology.

[8] Darwin CR (1877). The Different Forms of Flowers on Plants of the Same Species.

[9] Renner SS, Ricklefs RE (1995). Dioecy and its correlates in the flowering plants.. Amer J Bot.

[10] Renner SS, Won H (2001). Repeated evolution of dioecy from monoecy in Siparunaceae (Laurales).. Systematic Biology.

[11] Dorken ME, Barrett SCG (2004). Sex determination and the evolution of dioecy from monoecy in *Sagittaria latifolia* (Alismataceae).. Proceedings of the Royal Society of London B.

[12] Charlesworth B, Charlesworth D (1978). A model for the evolution of dioecy and gynodioecy.. Amer Nat.

[13] Westergaard M (1958). The mechanism of sex determination in dioecious plants.. Adv Genet.

[14] Charlesworth D, Charlesworth B, Marais G (2005). Steps in the evolution of heteromorphic sex chromosomes.. Heredity.

[15] Nicolas M, Marais G, Hykelova V, Janousek B, Laporte V (2005). A gradual process of recombination restriction in the evolutionary history of the sex chromosomes in dioecious plants.. PLoS Biol.

[16] Bergero R, Forrest A, Kamau E, Charlesworth D (2007). Evolutionary strata on the X chromosomes of the dioecious plant Silene latifolia: evidence from new sex-linked genes.. Genetics.

[17] Filatov DA, Moneger F, Negrutiu I, Charlesworth D (2000). Low variability in a Y-linked plant gene and its implications for Y-chromosome evolution.. Nature.

[18] Laporte V, Filatov DA, Kamau E, Charlesworth D (2005). Indirect evidence from DNA sequence diversity for genetic degeneration of Y-chromosome in dioecious species of the plant *Silene*: the *SlY4*/*SlX4* and *DD44*-X/*DD44*-Y gene pairs.. J Evol Biol.

[19] Matsunaga S, Isono E, Kejnovsky E, Vyskot B, Dolezel J (2003). Duplicative transfer of a MADS box gene to a plant Y chromosome.. Mol Biol Evol.

[20] Rautenberg A, Hathaway L, Oxelman B, Prentice HC (2010). Geographic and phylogenetic patterns in Silene section *Melandrium* (Caryophyllaceae) as inferred from chloroplast and nuclear DNA sequences.. Molecular Phylogenetics and Evolution.

[21] Heilbuth JC (2000). Lower species richness in dioecious clades.. American Naturalist.

[22] Filatov DA (2005). Evolutionary history of Silene latifolia sex chromosomes revealed by genetic mapping of four genes.. Genetics.

[23] Zluvova J, Lengerova M, Markova M, Hobza R, Nicolas M (2005). The inter-specific hybrid Silene latifolia×S. viscosa reveals early events of sex chromosome evolution.. Evol Dev.

[24] Mrackova M, Nicolas M, Hobza R, Negrutiu I, Moneger F (2008). Independent origin of sex chromosomes in two species of the genus Silene.. Genetics.

[25] Popp M, Oxelman B (2004). Evolution of a RNA polymerase gene family in *Silene* (Caryophyllaceae) — incomplete concerted evolution and topological congruence among paralogues.. Systematic Biology.

[26] Oxelman B, Lidén M (1995). Generic boundaries in the tribe Sileneae (Caryophyllaceae) as iInferred from nuclear rDNA sequences.. Taxon.

[27] Oxelman B, Lidén M, Berglund D (1997). Chloroplast rps16 intron phylogeny of the tribe Sileneae (Caryophyllaceae).. Plant Systematics and Evolution.

[28] Oxelman B, Lidén M, Rabeler R, Popp M, Oxelman B (2001). A revised generic classification of the tribe Sileneae (Caryophyllaceae).. Nordic Journal of Botany.

[29] Fior S, Karis PO, Casazza G, Minuto L, Sala F (2006). Molecular phylogeny of the Caryophyllaceae (Caryophyllales) inferred from chloroplast matK and nuclear rDNA ITS sequences.. American Journal of Botany.

[30] Erixon P, Oxelman B (2008). Reticulate or tree-like chloroplast DNA evolution in Sileneae (Caryophyllaceae)?. Molecular Phylogenetics and Evolution.

[31] Frajman B, Oxelman B (2007). Reticulate phylogenetics and phytogeographical structure of Heliosperma (Sileneae, Caryophyllaceae) inferred from chloroplast and nuclear DNA sequences.. Molecular Phylogenetics and Evolution.

[32] Desfeux C, Lejeune B (1996). Systematics of euromediterranean *Silene* (Caryophyllaceae) - evidence from a phylogenetic analysis using ITS sequences.. Comptes Rendus de l'Academie des Sciences Serie III-Sciences de la Vie-Life Sciences.

[33] Kejnovsky E, Kubat Z, Hobza R, Lengerova M, Sato S (2006). Accumulation of chloroplast DNA sequences on the Y chromosome of Silene latifolia.. Genetica.

[34] Mower J, Touzet P, Gummow J, Delph L, Palmer J (2007). Extensive variation in synonymous substitution rates in mitochondrial genes of seed plants.. BMC Evol Biol.

[35] Sloan D, Oxelman B, Rautenberg A, Taylor D (2009). Phylogenetic analysis of mitochondrial substitution rate variation in the angiosperm tribe Sileneae.. BMC Evol Biol.

[36] Kapralov MV, Filatov DA (2007). Molecular adaptation during adaptive radiation in the Hawaiian endemic genus *Schiedea*.. PLoS ONE.

[37] Muir G, Filatov D (2007). A selective sweep in the Chloroplast DNA of dioecious *Silene* (Section Elisanthe).. Genetics.

[38] Erixon P, Oxelman B (2008). Whole-gene positive selection, elevated synonymous substitution rates, duplication, and indel evolution of the chloroplast clpP1 gene.. PLoS ONE.

[39] Bergero R, Qiu S, Forrest A, Borthwick H, Charlesworth D

[40] Atanassov I, Delichere C, Filatov DA, Charlesworth D, Negrutiu I (2001). Analysis and evolution of two functional Y-linked loci in a plant sex chromosome system.. Mol Biol Evol.

[41] Jeanmougin F, Thompson JD, Gouy M, Higgins DG, Gibson TJ (1998). Multiple sequence alignment with Clustal X.. Trends Biochem Sci.

[42] Hall TA (1999). BioEdit: a user-friendly biological sequence alignment editor and analysis program for Windows 95/98/NT.. Nucleic Acids Symposium Series.

[43] Castresana J (2000). Selection of conserved blocks from multiple alignments for their use in phylogenetic analysis.. Molecular Biology and Evolution.

[44] Talavera G, Castresana J (2007). Improvement of phylogenies after removing divergent and ambiguously aligned blocks from protein sequence alignments.. Systematic Biology.

[45] Roure B, Rodriguez-Ezpeleta N, Philippe H (2007). SCaFoS: a tool for selection, concatenation and fusion of sequences for phylogenomics.. BMC Evolutionary Biology.

[46] Leigh J, Susko E, Baumgartner M, Roger A (2008). Testing congruence in phylogenomic analysis.. Systematic Biology.

[47] Guindon S, Gascuel O (2003). A simple, fast, and accurate algorithm to estimate large phylogenies by maximum likelihood.. Syst Biol.

[48] Guindon S, Lethiec F, Duroux P, Gascuel O (2005). PHYML Online — a web server for fast maximum likelihood-based phylogenetic inference.. Nucleic Acids Research.

[49] Filatov DA, Charlesworth D (2002). Substitution rates in the X- and Y-linked genes of the plants, Silene latifolia and S. dioica.. Mol Biol Evol.

[50] Marais GA, Nicolas M, Bergero R, Chambrier P, Kejnovsky E (2008). Evidence for degeneration of the Y chromosome in the dioecious plant Silene latifolia.. Curr Biol.

[51] Qiu S, Bergero R, Forrest A, Kaiser VB, Charlesworth D (2010). Nucleotide diversity in Silene latifolia autosomal and sex-linked genes.. Proc Biol Sci.

[52] Posada D (2008). jModelTest: phylogenetic model averaging.. Mol Biol Evol.

[53] Posada D, Buckley TR (2004). Model selection and model averaging in phylogenetics: advantages of akaike information criterion and bayesian approaches over likelihood ratio tests.. Syst Biol.

[54] Criscuolo A, Berry V, Douzery E, Gascuel O (2006). SDM: a fast distance-based approach for (super) tree building in phylogenomics.. Systematic Biology.

[55] Gascuel O (1997). BIONJ: an improved version of the NJ algorithm based on a simple model of sequence data.. Molecular Biology and Evolution.

[56] Edwards SV, Liu L, Pearl DK (2007). High-resolution species trees without concatenation.. Proceedings of the National Academy of Sciences of the USA.

[57] Rannala B, Yang Z (2003). Bayes estimation of species divergence times and ancestral population sizes using DNA sequences from multiple loci.. Genetics.

[58] Linder C, Rieseberg L (2004). Reconstructing patterns of reticulate evolution in plants.. American Journal of Botany.

[59] Cummings M, Neel M, Shaw K (2008). A genealogical approach to quantifying lineage divergence.. Evolution.

[60] Liu L, Yu L (2010). Phybase: an R package for species tree analysis.. Bioinformatics.

[61] Liu L, Yu L, Pearl D, Edwards S (2009). Estimating species phylogenies using coalescence times among sequences.. Syst Biol.

[62] Jobb G, Haeseler Av, Strimmer K (2004). TREEFINDER: a powerful graphical analysis environment for molecular phylogenetics.. BMC Evolutionary Biology.

[63] Pagel M, Meade A, Barker D (2004). Bayesian estimation of ancestral character states on phylogenies.. Systematic Biology.

[64] Jürgens A, Witt T, Gottsberger G (2002). Pollen grain numbers, ovule numbers and pollen-ovule ratios in Caryophylloideae: correlation with breeding system, pollination, life form, style number, and sexual system.. Sex Plant Reprod.

[65] Matsunaga S, Yagisawa F, Yamamoto M, Uchida W, Nakao S (2002). LTR retrotransposons in the dioecious plant *Silene latifolia*.. Genome.

[66] Touzet P, Delph L (2009). The effect of breeding system on polymorphism in mitochondrial genes of *Silene*.. Genetics.

[67] Yang Z (2007). PAML 4: Phylogenetic Analysis by Maximum Likelihood.. Molecular Biology and Evolution.

[68] Markova M, Michu E, Vyskot B, Janousek B, Zluvova J (2007). An interspecific hybrid as a tool to study phylogenetic relationshipsin plants using the GISH technique.. Chromosome Research.

[69] Stamatakis A (2006). RAxML-VI-HPC: maximum likelihood-based phylogenetic analyses with thousands of taxa and mixed models.. Bioinformatics.

[70] Popp M, Oxelman B (2001). Inferring the history of the polyploid *Silene aegaea* (*Caryophyllaceae*) using plastid and homoeologous nuclear DNA sequences.. Molecular Phylogenetics and Evolution.

[71] Li G, Steel M, Zhang L (2008). More taxa are not necessarily better for the reconstruction of ancestral character states.. Systematic Biology.

[72] Salisbury B, Kim J (2001). Ancestral state estimation and taxon sampling density.. Syst Biol.

[73] Mabberley D (1997). The Plant Book.

[74] Lidén M, Popp M, Oxelman B (2001). A revised generic classification of the tribe Sileneae (Caryophyllaceae).. Nordic Journal of Botany.

[75] Bergero R, Charlesworth D, Filatov DA, Moore RC (2008). Defining regions and rearrangements of the Silene latifolia Y chromosome.. Genetics.

[76] Rautenberg A, Filatov D, Svennblad B, Heidari N, Oxelman B (2008). Conflicting phylogenetic signals in the SlX1/Y1 gene in *Silene*.. BMC Evol Biol.

