# Supplementary figures and images for: Multiple Nuclear Gene Phylogenetic Analysis of the Evolution of Dioecy and Sex Chromosomes in the Genus Silene
Source: PLoS One. 2011 Aug 10;6(8):e21915. doi: 10.1371/journal.pone.0021915 (PMC3154253; doi:10.1371/journal.pone.0021915)

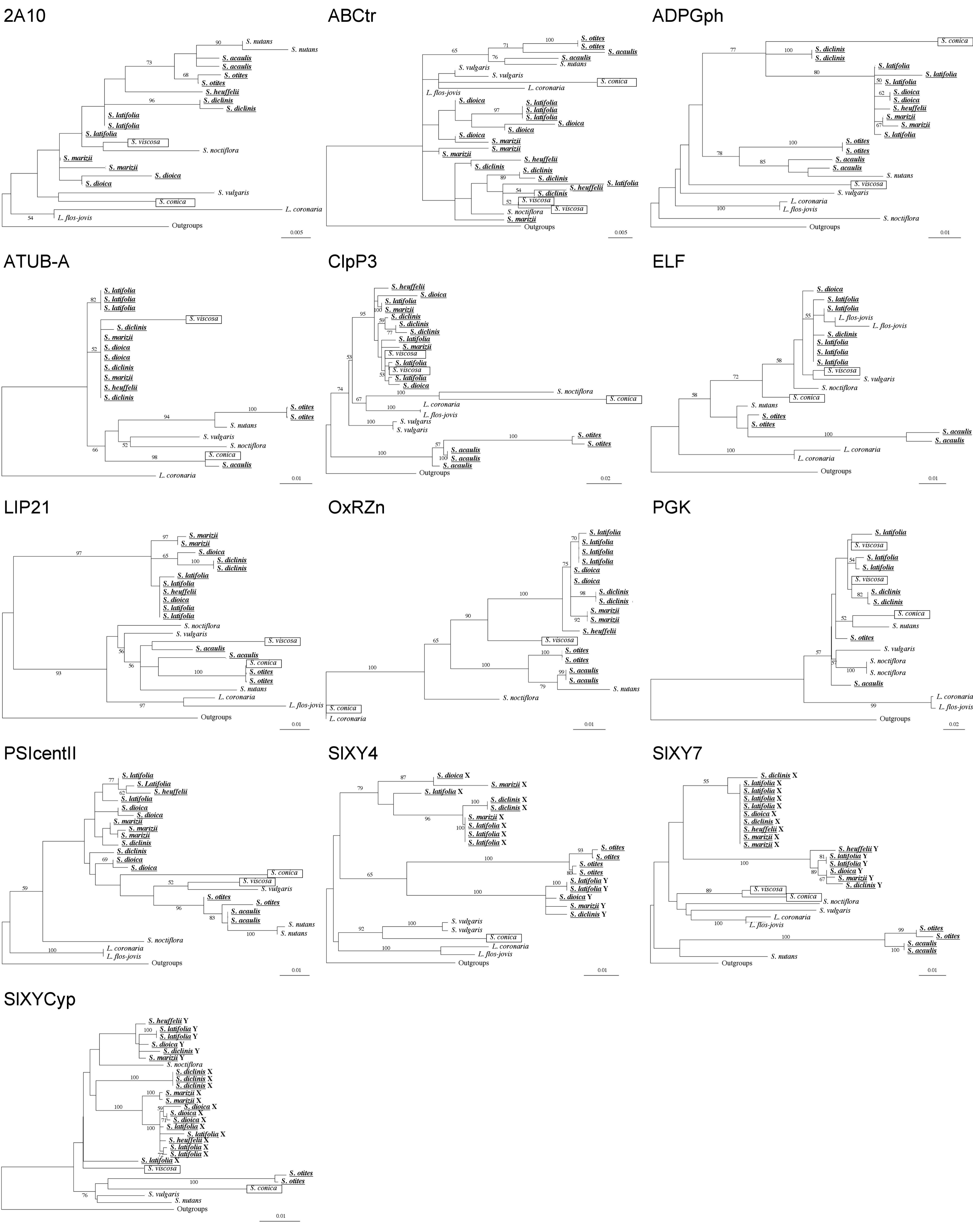

Supplement: Figure S1 — Individual gene trees for our 13 genes. Trees were obtained with PhyML (see Material and Methods) with 100 non-parametric bootstrap replicates (only values >50% are shown). Note that some of the sequences were excluded from the further analyses, as follows: cDNA sequences from S. latifolia for ABCtr, PGK, SlXY4 and SlXYCyp, whose sequence lengths were too different, and the Petrocoptis sequence of OxRZn because of doubts of the orthology of the sequence. For each gene, there is one outgroup sequence. As noted in Table S2 and footnote 4 of Table S3, the outgroup species used to root the tree is usually Petrocoptis hispanica; this sequence was the outgroup for 8 of the 13 trees shown, including all the X-linked genes, but, for PGK, a Dianthus sequence was used, and Lychnis for ATUB-A and OxRZn. (TIFF) [file pone.0021915.s001.tif]

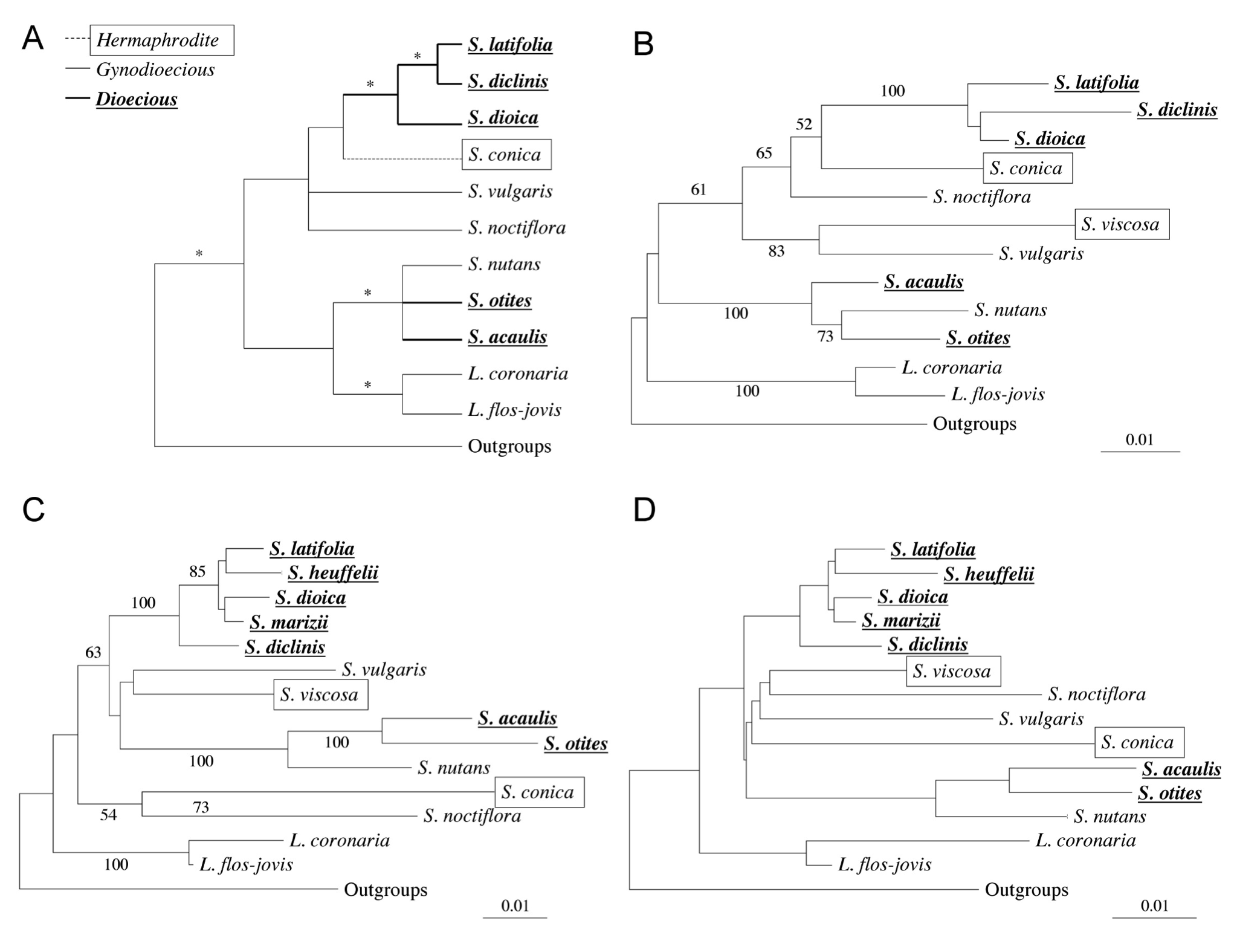

Supplement: Figure S2 — Other inferred phylogenies of the Silene species studied. For details about sequences and species, see Table S3. Only bootstrap values >50% are shown. A) Maximum parsimony tree of the ITS sequences from 12 species from [4] that were also studied by ourselves. The different mating systems are indicated, and stars indicate bootstrap values larger than 50%. B) Maximum likelihood tree (PhyML) of the ITS sequences from 13 of our species. C) Maximum likelihood tree of 7 autosomal genes concatenated by Concaterpillar (see Table S4) with all the species (except outgroups for 3 genes: 2A10, ClpP3 and ELF, see main text and Table S4) obtained with PhyML. Identical results were found with 4 or 8 categories of sites. In trees C and D, and also those in Figure 1 of the main text, only X sequences were included for the sex-linked genes. D) Dataset as in C, but the tree was obtained by combining all the 7 gene trees using the SDM method. In C, S. conica and S. noctiflora diverge early, but in D they group with S. vulgaris and S. viscosa. (TIFF) [file pone.0021915.s002.tif]

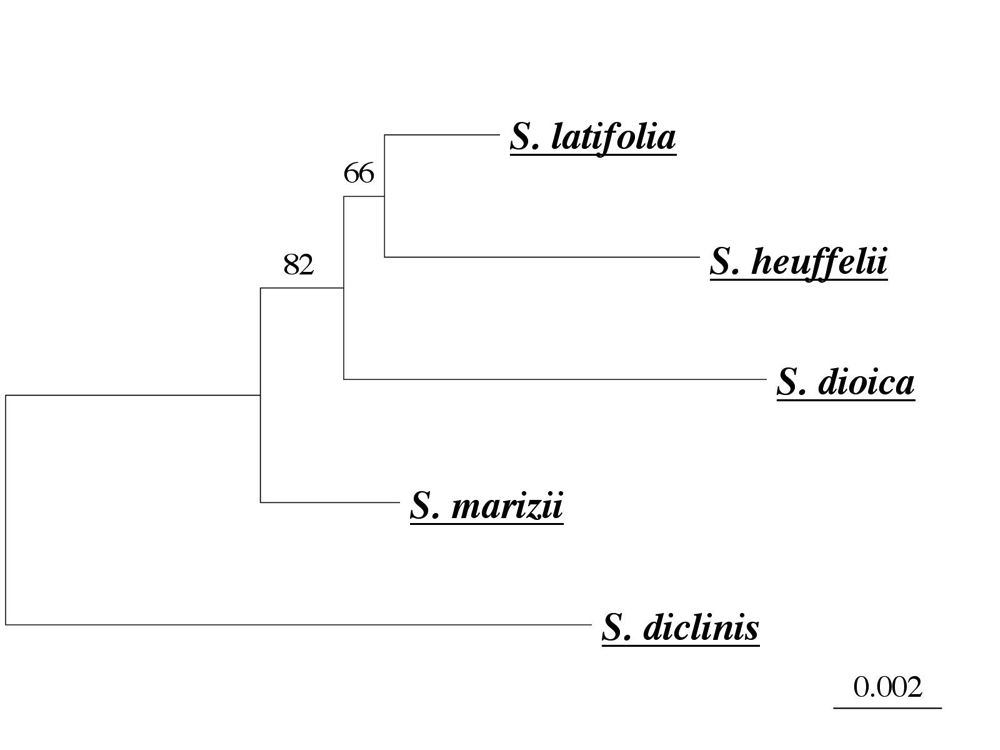

Supplement: Figure S3 — Tree for dioecious species, rooted using S. diclinis (as this species is early diverging in all trees in Figure 1 and Figure S2). The tree was obtained by PhyML on the concatenate of all genes without Gblocking. Values indicate the results of non-parametric bootstrapping with 100 replicates (only values >50% are shown). (TIFF) [file pone.0021915.s003.tif]

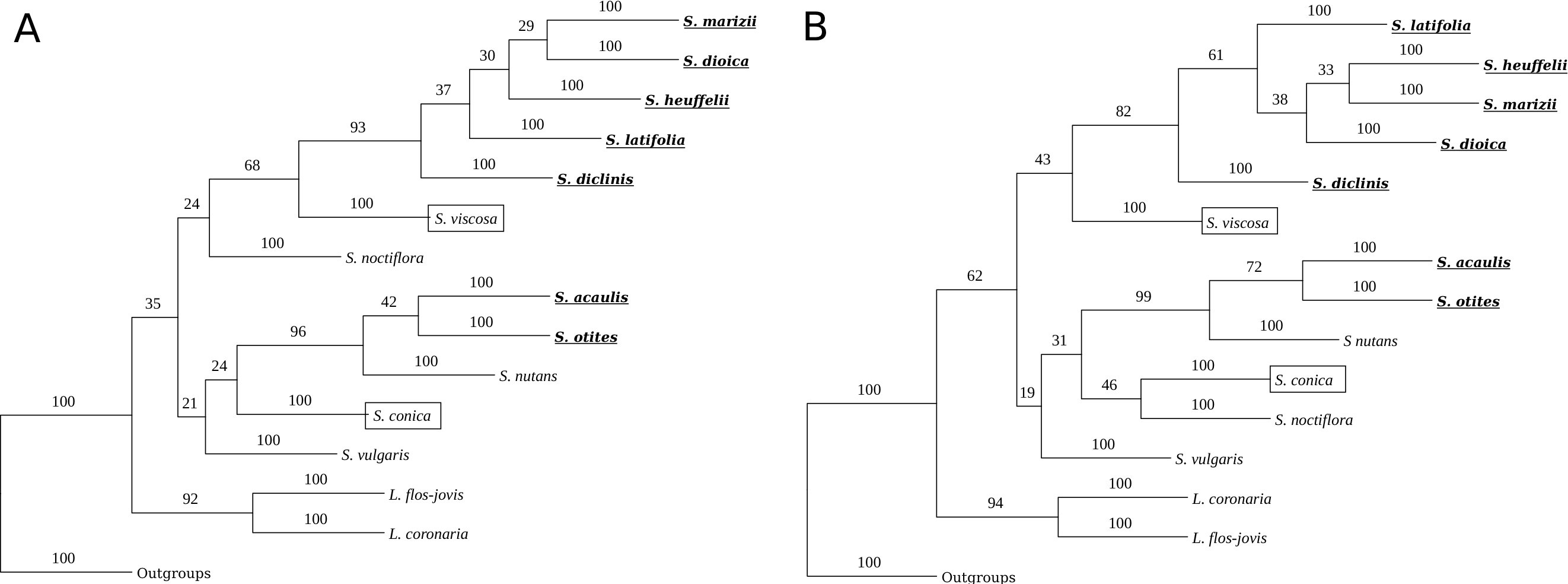

Supplement: Figure S4 — Estimated species trees, using all genes except for LIP21 (12 loci). A) Tree using STAR. B) Tree using STEAC. In both cases, the branch lengths are proportional to the bootstrap support, which is given as a percentage at each branch. As for the individual locus trees, the outgroup species was Petrocoptis hispanica for all genes except PGK, where a Dianthus sequence was used, and ATUB-A and OxRZn where no outgroups were available. (TIFF) [file pone.0021915.s004.tif]
