# Supplementary material for: Multiple Nuclear Gene Phylogenetic Analysis of the Evolution of Dioecy and Sex Chromosomes in the Genus Silene
Source: PLoS One. 2011 Aug 10;6(8):e21915. doi: 10.1371/journal.pone.0021915 (PMC3154253; doi:10.1371/journal.pone.0021915)
Supplement: Table S1 — Provenance and voucher details of plants used to assess autosomal and sex-linked gene phylogenies in Silene . (DOC) [file pone.0021915.s005.doc]

**Table S1.** Provenance and voucher details of plants used to assess autosomal and sex-linked gene phylogenies in *Silene*. Voucher specimens are not available for four plants. For one *Silene acaulis* plant (from Spain), and the *Lychnis flos-jovis* and *Dianthus* plants died before specimens could be obtained, and for *S. heuffelii*, our sample was a DNA sample donated by D.A. Filatov.

| **Species** | **Plant** | **Collection Locality** | **Voucher (E)** | **Breeding system** |
| --- | --- | --- | --- | --- |
| *Silene latifolia* Poir. | 98E4.9 ♀ | Scotland: Dalkeith | E00243338 | Dioecious |
| *Silene latifolia* Poir. | 99K4.5 ♂ | England: Sussex | E00243339 | Dioecious |
| *Silene latifolia* Poir. | 99L10.1 ♂ | France: Canche | E00243340 | Dioecious |
| *Silene heuffelii* Soó | IL5E ♂ | Romania | Not available | Dioecious |
| *Silene marizii* Samp. | MG0713 ♀ | Portugal: Mangualde | E00243341 | Dioecious |
| *Silene marizii* Samp. | PT0616 ♂ | Portugal: Porto | E00243342 | Dioecious |
| *Silene dioica* (L.) Clairv. | 99K22.7 ♀ | France: Correze | E00243343 | Dioecious |
| *Silene dioica* (L.) Clairv. | 9.1 ♂ |  | E00243344 | Dioecious |
| *Silene diclinis* (Lag.) M. Laínz | A2000.13.4 ♀ | Spain: Valencia | E00243345 | Dioecious |
| *Silene diclinis* (Lag.) M. Laínz | A2000.13.10 ♂ | Spain: Valencia | E00243346 | Dioecious |
| *Silene noctiflora* L. | A2000.14.1 | Lund, Sweden | E00243347 | Hermaphrodite, gynodioecious/gynomonoecious |
| *Silene vulgaris* (Moench) Garcke | 99K11.2 | England: Sussex | E00243348 | Gynodioecious/gynomonoecious |
| *Silene conica* [L.](http://es.wikipedia.org/wiki/Carlos_Linneo) | 99L7.1 | France: Canche | E00243349 | Hermaphrodite |
| *Silene viscosa* Pers. | D2004.1.1 | Sweden: Kallskär, Bråviken | E00243350 | Hermaphrodite |
| *Silene otites* Sm. | K2000.3.3 ♀ | Germany: Erfurt Nord | E00243351 | Dioecious, sub-dioecious [[1]](#footnote-2) |
| *Silene otites* Sm. | K2000.4.10 ♂ | Germany: Erfurt Nord | E00243352 | Dioecious, sub-dioecious |
| *Silene acaulis* ([L.](http://en.wikipedia.org/wiki/Carolus_Linnaeus)) [Jacq.](http://en.wikipedia.org/wiki/Nikolaus_Joseph_von_Jacquin) | RBGE19850884 | Scotland: Lewis | E00228616 | Gynodioecious or dioecious |
| *Silene acaulis* ([L.](http://en.wikipedia.org/wiki/Carolus_Linnaeus)) [Jacq.](http://en.wikipedia.org/wiki/Nikolaus_Joseph_von_Jacquin) | RBGE19951843 | Spain | Not available | Gynodioecious or dioecious |
| *Silene nutans* [L.](http://en.wikipedia.org/wiki/Carolus_Linnaeus) | 99O.1.1 | France | E00243353 [[2]](#footnote-3) | Gynodioecious/gynomonoecious |
| *Lychnis coronaria* ([L.](http://en.wikipedia.org/wiki/Carl_Linnaeus)) [Clairv.](http://en.wikipedia.org/wiki/Joseph_Philippe_de_Clairville) | RBGE19632698 | Turkey: Zonguldak | E00228576 | Gynodioecious/gynomonoecious |
| *Lychnis flos-jovis* Desr. | RBGE19271025 | Germany: Bavaria (Univ. Munich Alpengarten) | Not available | Gynodioecious/gynomonoecious |
| *Petrocoptis hispanica* Pau. | RBGE19902058 | Spain: Huesca | E00228584 | Hermaphrodite |
| *Dianthus [[3]](#footnote-4)* | Cultivar | No information | Not available | Hermaphrodite |

1. See text [↑](#footnote-ref-2)
2. Accession number 1988138.1 from Jardin Botanique, Nantes 44000, France [↑](#footnote-ref-3)
3. Used as outgroup for the *PGK* gene only [↑](#footnote-ref-4)
