# Supplementary material for: Multiple Nuclear Gene Phylogenetic Analysis of the Evolution of Dioecy and Sex Chromosomes in the Genus Silene
Source: PLoS One. 2011 Aug 10;6(8):e21915. doi: 10.1371/journal.pone.0021915 (PMC3154253; doi:10.1371/journal.pone.0021915)
Supplement: Table S2 — Primers used to amplify genomic DNA of the genes studied, lengths of the sequences studied, and the outgroup used. (DOC) [file pone.0021915.s006.doc]

**Table S2.** Primers used to amplify genomic DNA of the genes studied, lengths of the sequences studied, and the outgroup used. For the sex-linked genes used in our analyses, the references to the original papers are indicated as footnotes.

|  |  | Primers for sequencing [[1]](#footnote-2) | |  | Sequence lengths (bp) | |  | |
| --- | --- | --- | --- | --- | --- | --- | --- | --- |
| Gene names | Accession numbers of *A. thaliana* sequence and putative functions, where known | Primer F | Primer R | Annealing temp. (ºC) | Coding | Non-coding | Outgroup | |
| Autosomal | in the dioecious species |  |  |  |  |  | |  |
| ABCtr | ABC transporter-like protein: AT3G54540 | 5´-CGACTCCATCCTGACC-3´ | 5´-GCTCCTCCTTGTATTCC-3´ | 52 | 332 | 0 | Petrocoptis | |
| ATUB | α tubulin: AT1G64740, AT5G19770, AT4G14960, AT1G50010, AT1G04820, AT5G19780 | 5´-TGCCCCCGTCATCTCTG-3´ | 5´-ACCTTCCTCCATACCCTCAC-3´ | 56 | 433 | 0 | Lychnis | |
| ADPGph | Glucose-1-phosphate adenylyltransferase, large subunit (ADP-glucose pyrophosphorylase): At2g21590, AT4G39210, AT1G27680 | 5´-TCTTGACTCTGGGGTAG-3´ | 5´-ATGACTGTGATGCCTGAT-3´ | 48 | 121 | 275 | Petrocoptis | |
| 2A10 | Non-intrinsic ABC protein 4: AT1G03900 | 5´-AAACACCATCACCATCATTCC-3´ | 5´-CGAAATAACGGCTAGAATCG-3´ | 52 | 262 | 0 | Petrocoptis | |
| ClpP3 | ATP-dependent Clp protease proteolytic subunit: AT1G66670 | 5´-CGTTTCTTCTCGCTTCTG-3´ | 5´-TATTGTATGCCCAATTCAA-3´ | 50 | 294 | 1060[[2]](#footnote-3) | Petrocoptis | |
| ELF | Eukaryotic elongation factor 1α: AT1G07920, AT1G07930, AT1G07940, AT5G60390, AT1G35550 | 5´-TAACGGTTATGCCCCAGTTC-3´ | 5´-GACTCCAACAGCAACGGTCT-3´ | 56 | 360 | 0 | Dianthus | |
| LIP21 | Lipoyltransferase: AT4G31050, AT1G47580 | 5´-GTCTCACATTGGCTTGC-3´ | 5´-GAACATCTCCGAACTACC-3´ | 50 | 198 | 212 | Petrocoptis | |
| OxRZn | Oxidoreductase, zinc-binding dehydrogenase family: AT1G23740 | 5´-TCCATCACTCTCTTCCACA-3´ | 5´-TTCATAGGCGGTCTCAAT-3´ | 52 | 381 | 0 | Lychnis (see text) | |
| PSIcentII | Photosystem I reaction center subunit II precursor–related: AT1G03130, AT4G02770 | 5´-ATCCATCAACTCCGTCTC-3´ | 5´-ACCAACACCTTCTCTTCC-3´ | 51 | 330 | 0 | Petrocoptis | |
| PGK | Phosphoglycerate kinase: AT1G79550, AT3G12780, AT1G56190 | 5´-CAATAAGTTTGCTGCTGAT-3´ | 5´-ACTGTACCGGCCTCTGCA-3´ | 48 | 221 |  | Dianthus | |
| Sex-linked | in the dioecious species |  |  |  |  |  |  | |
| SlX4[[3]](#footnote-4) | Putative fructose-2,6-bisphosphatase | 11+ from [[4]](#footnote-5) | 5´-AATTACCGAAGACAGTAAAGCGTC-3´ | > 58 | 699 | 0 | Petrocoptis | |
| SlY4^3^ | Putative fructose-2,6-bisphosphatase | 10 from ^4^ | 5´-AATCACACAGTTGATCTCATTTTCC-3´ | > 55 | 699 | 0 | Petrocoptis | |
| SlX7[[5]](#footnote-6) | Unknown protein: At5G48020 | Y7stF: 5´-TGTTACAGACCGCACTCTCG-3´ | Y7stR: 5´-ACGGAAAGCTCCTTTTCAAC-3´ | 55 | 199 |  | Petrocoptis | |
| SlY7^5^ | At5G48020 | Y7stF, as above | Y7r1: 5´-ACGGCAAGCTCTTTTTCAACGAAC-3´ | 55 | 199 |  | Petrocoptis | |
| SlCyp-XY^5^ | Cyclophilin: At3G44600 | 5’-CAGAGACAGCACCCCAACC-3’ | 5’-CTTCTGGGTAAAGCTTCATGTG-3’ | 55 | 480 | 638 | Petrocoptis | |

1. The primers used for sequencing were designed using Primer 3 software [77] [↑](#footnote-ref-2)
2. 212 bp, excluding a region present only in *S. acaulis*, *S. otites*, *Lychnis* and *Petrocoptis* [↑](#footnote-ref-3)
3. Ref. 40 [↑](#footnote-ref-4)
4. Ref. 18 [↑](#footnote-ref-5)
5. Ref. 16 [↑](#footnote-ref-6)
