# Supplementary material for: Multiple Nuclear Gene Phylogenetic Analysis of the Evolution of Dioecy and Sex Chromosomes in the Genus Silene
Source: PLoS One. 2011 Aug 10;6(8):e21915. doi: 10.1371/journal.pone.0021915 (PMC3154253; doi:10.1371/journal.pone.0021915)
Supplement: Table S3 — List of species and sequences. (DOC) [file pone.0021915.s007.doc]

**Table S3.** List of species and sequences.

| Species [[1]](#footnote-2) | Breeding systems [[2]](#footnote-3) | AUTOSOMAL GENES | | | | | | | | | |  | SEX-LINKED | | |
| --- | --- | --- | --- | --- | --- | --- | --- | --- | --- | --- | --- | --- | --- | --- | --- |
|  |  | ***ABCtr*** | ***ATUB-A*** | *2A10* | ***ClpP3*** | ***ELF*** | ***LIP21*** | ***OxRZn*** | ***PSIcentII*** | ***PGK*** | ***ADPGph*** |  | ***SlXY4*** | ***SlXY7*** | ***SlCy*XY*p*** |
| S. acaulis | *D* | 2 | 1 | 2 | 3 | 2 | 2 | 2 | 2 | 1 | 2 |  | 0 | 2 | 2 |
| S. diclinis | *D* | 4 | 3 | 2 | 3 | 1 | 2 | 2 | 2 | 2 | 2 |  | 3 | 3 | 4 |
| ***S. dioica*** | *D* | 4 | 2 | 2 | 2 | 1 | 2 | 2 | 4 | 0 | 2 |  | 2 | 3 | 4 |
| *S. heuffelii* | *D* | 2 | 1 | 1 | 1 | 0 | 1 | 1 | 1 | 0 | 1 |  | 0 | 2 | 2 |
| S. latifolia | *D* | 3 | 3 | 3 | 4 | 5 | 4 | 3 | 3 | 3 | 4 |  | 6 | 5 | 6 |
| *S. marizii* | *D* | 4 | 2 | 2 | 2 | 0 | 2 | 2 | 3 | 0 | 2 |  | 3 | 3 | 3 |
| S. otites | *D* | 2 | 2 | 2 | 2 | 2 | 2 | 2 | 2 | 1 | 2 |  | 4 | 2 | 1 |
| L. coronaria | *G* | 1 | 1 | 1 | 1 | 2 | 1 | 1 | 1 | 1 | 1 |  | 1 | 2 | 0 |
| ***L. flos-jovis*** | *G* | 1 | 0 | 1 | 1 | 2 | 1 | 0 | 1 | 1 | 1 |  | 1 | 2 | 0 |
| S. noctiflora | G | 1 | 1 | 1 | 1 | 1 | 1 | 1 | 1 | 2 | 1 |  | 0 | 1 | 1 |
| *S. nutans* | *G* [[3]](#footnote-4) | 1 | 1 | 2 | 0 | 1 | 1 | 1 | 2 | 1 | 1 |  | 0 | 1 | 0 |
| S. vulgaris | *G* ^3^ | 2 | 1 | 1 | 2 | 1 | 1 | 0 | 1 | 0 | 1 |  | 2 | 1 | 1 |
| S. conica | *H* | 1 | 1 | 1 | 1 | 1 | 1 | 1 | 1 | 1 | 1 |  | 1 | 1 | 1 |
| S. viscosa | H | 2 | 1 | 1 | 2 | 1 | 1 | 1 | 1 | 2 | 1 |  | 0 | 1 | 1 |
| Outgroups [[4]](#footnote-5) |  | 1 | 0 | 1 | 1 | 2 | 2 | 0 | 1 | 1 | 1 |  | 1 | 1 | 1 |
| No. sites [[5]](#footnote-6) |  | 332 | 433 | 262 | 1354 | 360 | 410 | 381 | 330 | 268 | 446 |  | 699 | 354 | 1118 |
| No. sites after cleaning [[6]](#footnote-7) |  | 332 | 433 | 262 | 491 | 249 | 323 | 351 | 330 | 223 | 413 |  | 699 | 341 | 1090 |

1. Species included in the analyses in Table S4 are in bold. [↑](#footnote-ref-2)
2. D = dioecious species, G = gynodioecious, H = hermaphroditic. Note uncertainties about *S. noctiflora* and the two *Lychnis* breeding systems (see text). [↑](#footnote-ref-3)
3. Gynomonoecious plants are present. [↑](#footnote-ref-4)
4. Outgroups are *Petrocoptis* sequences, except for *PGK*, where a *Dianthus* sequence was used. [↑](#footnote-ref-5)
5. Number of sites in alignment [↑](#footnote-ref-6)
6. Number of sites after cleaning the alignment with Gblocks (see Methods) [↑](#footnote-ref-7)
