# Supplementary material for: Multiple Nuclear Gene Phylogenetic Analysis of the Evolution of Dioecy and Sex Chromosomes in the Genus Silene
Source: PLoS One. 2011 Aug 10;6(8):e21915. doi: 10.1371/journal.pone.0021915 (PMC3154253; doi:10.1371/journal.pone.0021915)
Supplement: Table S4 — Results of tests for combining alignments using Concaterpillar. (DOC) [file pone.0021915.s008.doc]

**Table S4.** Results of tests for combining alignments using Concaterpillar. Initial analysis found 3 blocks of genes (4 single-copy genes agree with the ITS phylogeny, 3 genes differ mainly in the position of the outgroup species, and a single gene, *LIP21*, differ from both these blocks); excluding outgroups yielded one block with all genes except *LIP21*, which we therefore excluded for subsequent analyses, as well as the outgroup sequences for the three genes in the second block above. We combined the data to build a tree, using either: the concatenated alignments (employing PhyML with the GTR model of sequence evolution and a gamma distribution (to allow rate differences among sites, Figure S2C), or a supertree (using the individual gene trees obtained using PhyML, Figure S2D).

|  | **Number of concatenates** | Genes included in the concatenates **[[1]](#footnote-2)** | **Significance level** |
| --- | --- | --- | --- |
| 11 species ^2^  with outgroups | 3 | 1: *ITS*, *ABCtr*, *ADPGph*, *PSIcentII*, *SLXY7*  2: *2A10*, *clpP3*, *ELF*  3: *LIP21* | 0.00231 |
| 11 species  without outgroups | 2 | 1: *ITS*, *2A10*, *ABCtr*, *SLXY7*, *ADPGph*, *PSIcentII*, *ELF*, *clpP3*  2: *LIP21* | 0.00402 |

1. “Conservative group” (excludes the autosomal genes *ATUB-A*, *OxRZn* and *PGK*, and X-linked *SlXY4* and *SlCyp-*XY), See Material and Methods for details

   ^2^ Species included in the analysis are in bold in Table S3*.* [↑](#footnote-ref-2)
