# Supplementary material for: Multiple Nuclear Gene Phylogenetic Analysis of the Evolution of Dioecy and Sex Chromosomes in the Genus Silene
Source: PLoS One. 2011 Aug 10;6(8):e21915. doi: 10.1371/journal.pone.0021915 (PMC3154253; doi:10.1371/journal.pone.0021915)
Supplement: Text S1 — Discussion on the differences in branch length in our trees. (RTF) [file pone.0021915.s009.rtf]

Text S1.

Single trees from concatenated sequences of several different genes are sensitive to differences in evolutionary rates. Our species indeed probably have different substitution rates (indicated by branch length differences in Figures 1, S1 and S2). Also, our genes include different amounts of coding regions (the three codon positions are known to evolve differently from one other, and from intron sites, see [78]). However, PhyML analyses with 8 site categories (to better capture rate heterogeneity) yielded exactly the same tree as with 4 categories (Figure S2C). Thus, rate heterogeneity among sites is probably less important than among species. S. noctiflora and S. conica are fast-evolving species, which concatenation deals with less well than SDM; their different locations in trees could thus result from long-branch attraction, a well-known artefact in phylogenetics.
