# Supplementary material for: Multiple Nuclear Gene Phylogenetic Analysis of the Evolution of Dioecy and Sex Chromosomes in the Genus Silene
Source: PLoS One. 2011 Aug 10;6(8):e21915. doi: 10.1371/journal.pone.0021915 (PMC3154253; doi:10.1371/journal.pone.0021915)
Supplement: Text S2 — Methods and results for the STAR and STEAC analysis. (RTF) [file pone.0021915.s010.rtf]

Text S2.

We used this approach in preference to the Bayesian method, BEST [56], to estimate the species tree, because BEST is computationally intensive, and is sensitive to missing sequences of genes for some of the species. With our data, it did not converge even after 6000 million iterations. 
We thus used methods based on the average of coalescence times or ranks [61]. The calculation of averages is very fast, and is not biased by missing data (although the estimates deteriorate). The two methods we employed are available in the R package phybase [60]: STAR is based on the average ranks of coalescence, STEAC on average coalescence times [61]. Both methods take account of deep coalescences, but have only limited robustness to gene flow. 
For each gene, we generated 1000 bootstrap datasets, using the 2-stage bootstrap procedure involving sampling of genes and species [79]. For each bootstrap replicate, the gene tree was estimated with PhyML [47] using the default parameters. We then used the program consense in the PHYLIP package [80] to calculate the consensus tree for each method.
The LIP21 gene was identified by Concaterpillar as having an anomalous phylogeny (see main text). We therefore estimated species trees using bootstrap replicated gene trees, either including LIP21 (13 loci), or excluding this locus. Considering only branches with at least 50% boostrap support, the species trees constructed including LIP21 are no different from those shown. 
The results using the two methods are similar, mainly differing in the bootstrap support for some branches; topological differences between the two methods do not exceed 50% bootstrap support. When the genes evolve according to a molecular clock, STEAC should yield better results than STAR, while STAR is better when this assumption is violated [61]. As we observed substantial differences in the lengths of the gene trees, indicating (biologically plausible) variation in substitution rates, the STAR tree topology (Figure S4A) is probably the more reliable of the two. There is considerable support for the monophyly of Silene using the STEAC method (62%, Figure S4B), but less using STAR (35%, Supplementary Figure 4A). The support for S. nutans being the direct outgroup of S. acaulis and S. otites, as well as for S. diclinis as the closest outgroup of S. marizii, S. latifolia, S. dioica and S. heuffelii, is higher for STEAC, while the location of S. viscosa at the base of the dioecious clade that includes S. latifolia is more highly supported with STAR.
