# Supplementary material for: Multiple Nuclear Gene Phylogenetic Analysis of the Evolution of Dioecy and Sex Chromosomes in the Genus Silene
Source: PLoS One. 2011 Aug 10;6(8):e21915. doi: 10.1371/journal.pone.0021915 (PMC3154253; doi:10.1371/journal.pone.0021915)
Supplement: Reference S1 — (RTF) [file pone.0021915.s011.rtf]

References (76-80)


77.	Yang, Z., Among-site variation and its impact on phylogenetic analyses. Trends in Ecology and Evolution, 1996. 11: p. 367-372.
78.	Seo, T., Calculating bootstrap probabilities of phylogeny using multilocus sequence data. Mol. Biol. Evol., 2008. 25(5): p. 960-971.
79.	Felsenstein, J., PHYLIP (Phylogeny Inference Package) version 3.6, 2005, Distributed by the author.
80.	Rozen, S. and H. Skaletsky, Primer3 on the WWW for general users and for biologist programmers. Methods in Molecular Biology, 2000. 132: p. 365-386.
